# Childhood obesity in relation to sweet taste perception and dental caries – a cross-sectional multicenter study

**DOI:** 10.29219/fnr.v63.1682

**Published:** 2019-04-04

**Authors:** Heba Ashi, Guglielmo Campus, Gunilla Klingberg, Heléne Bertéus Forslund, Peter Lingström

**Affiliations:** 1 Department of Cariology, Institute of Odontology, Sahlgrenska Academy, University of Gothenburg, Gothenburg, Sweden; 2 Department of Public Health, Faculty of Dentistry, King Abdulaziz University, Jeddah, Saudi Arabia; 3 Klinik für Zahnerhaltung, Präventiv- und Kinderzahnmedizin, Zahnmedizinische Kliniken (ZMK), University of Bern, Switzerland and Department of Surgery, Microsurgery; 4 WHO Collaborating Centre for Epidemiology and Preventive Dentistry, Milan, Italy; 5 Department of Paediatric Dentistry, Faculty of Odontology, Malmö University, Malmö, Sweden; 6 Department of Internal Medicine and Clinical Nutrition, Institute of Medicine, Sahlgrenska Academy, University of Gothenburg, Gothenburg, Sweden

**Keywords:** dental caries, Italy, Mexico, Obesity, Saudi Arabia, taste perception

## Abstract

**Background:**

Obesity is a multifactorial disease that is increasing worldwide and is caused by different environmental and genetic factors, with an increase in the consumption of high-energy–containing food and a decrease in physical activity constituting two of the main reasons. Sweet taste perception may have an effect on the subject’s dietary choices and affect his or her predisposition to obesity.

**Objectives:**

The aim was to study the sweet taste perception and dental caries in relation to body mass index (BMI) in 13–15-year-old schoolchildren from three different countries and to compare the BMI among the countries.

**Design:**

The sweet taste perception level, determined as the sweet taste threshold and preference, was assessed in a total of 669 schoolchildren from Italy, Mexico and Saudi Arabia, examined in school settings. Height and weight were collected and BMI was calculated, after which the children were grouped as underweight, normal, overweight, and obese. For caries registration, the International Caries Detection and Assessment System and Decayed Missing Filled Surfaces indices were used.

**Results:**

A statistically significant difference was found for BMI among the children from the three countries (*p* < 0.001), with the highest mean found among Saudi children, followed by Mexican and Italian children. A statistically significant difference regarding sweet taste threshold when comparing the BMI groups was only found for Saudi Arabia (*p* < 0.01). No significant correlation was found between BMI and sweet taste threshold or preference and dental caries variables, respectively.

**Conclusions:**

BMI was found to differ between countries, with a further significant difference among the groups among the Saudi Arabia schoolchildren.

## Popular scientific summary

- BMI vary between 13–15-year-old children from Italy, Mexico and Saudi Arabia.
- Significant difference for sweet taste threshold for different BMI groups was found for children from Saudi Arabia.

## Introduction

Obesity is a multifactorial disease that is increasing worldwide and is currently of great concern, especially in low- and middle-income countries (1, 2). More than 42 million overweight children were reported in 2014, with the prevalence doubling from 1980 to 2014 (3). Obesity is the result of an imbalance in energy, where the consumption of energy exceeds that expended (4). Different cultural, environmental, genetic, and socioeconomic factors may determine a subject’s predisposition to obesity (2, 4, 5).

Diet is one of the main factors behind obesity in children (6). Among other elements, dietary intake and food choices are influenced by taste perception (7–9), which in turn influences the risk of developing obesity (10). As their age increases, obese subjects have been found to have less sensitive taste abilities, unlike individuals with normal weight in whom taste is more pronounced with age (10, 11).

A high preference for sweets during childhood is thought to prompt the choice of foods rich in calories (12, 13). In addition, children are also at higher risk for increased sweet consumption for other reasons, such as a lower cognitive ability, parental influence, and the association of sweet preference with growth (12, 14). However, sweet taste preference declines with age, as less energy usually is needed in the elderly (13). In addition, reducing the consumption of sugars can alter the perception of sweet (15). Moreover, another factor that has been found to alter the perception of a sweet solution is the color of the solution (16).

Childhood obesity is also known to have an effect on oral health both to periodontal disease (17) and to dental caries, as well as influencing salivary flow rate (18). All of this emphasizes the effect of obesity on children’s oral health.

The aim was to study the sweet taste perception and dental caries in relation to body mass index (BMI) in 13–15-year-old schoolchildren from three different countries and to compare the BMI among the countries. The study was based on the hypothesis that a correlation would be found between BMI and both sweet taste perception and dental caries. In addition, BMI mean value might differ between countries.

## Materials and Methods

### Subjects and study design

A cross-sectional study comprising 669 schoolchildren aged 13–15 years (220 Italian, 224 Mexican, and 225 Saudi Arabian) was conducted. Data were collected from three countries (Italy, Mexico, and Saudi Arabia) as part of a multicenter study with the aim of evaluating sweet taste perception in relation to different tested variables. Data on the relationship between sweet taste perception, diet and dental caries has been addressed in a previous paper (19, 20).

A list of all the children was drawn up from the chosen schools, and 1% of the population was selected. Schools were similar from a socioeconomic standpoint and students’ classes were as comparable as possible. A similar sample size for each area (=200) was chosen during the study design phase. After the survey for *post hoc* power, an analysis was calculated with a non-centrality parameter of 18, a critical χ^2^ of 7.81, and a power (1–β error probablity) of 0.96.

The study subjects were children aged 13–15 years from the participating schools, who had been living in the country from at least 6 years of age and were free of any systemic medical conditions. Children undergoing orthodontic treatment with fixed orthodontic appliances were excluded from the study, as well as children presenting with flu symptoms at the time of the examination.

Data collection was carried out at school visits. Data related to height and weight measurements were gathered, and a sweet taste perception assessment and a clinical examination of caries registration were performed. All measurements were carried out in the children’s classrooms. Calibration between examiners was performed prior to start of the study.

The study was approved by the responsible directorate of education and the ethics committee institute in each country (University of Sassari, Italy, ethical approval no.1073/L 23/07/2012; Secretary of Education of Veracruz, Mexico, ethical approval S.E.V. 30FIS0030Z; King Abdulaziz University, Jeddah, Saudi Arabia, ethical approval no. 029-12). Children who met the criteria in each country were voluntarily recruited and informed about the nature of the study. Informed consent was completed and signed by the children’s parents.

### Anthropometric measurements

Data on the height (cm) and weight (kg) of the children were obtained using portable scales. BMI was then calculated for each subject (BMI = body weight divided by the square of height). The children were asked to remove their shoes, socks, and any heavy clothing prior to measurement. BMI was categorized as underweight, normal, overweight, or obese (into quartiles), depending on the mean age and gender of each participant, following the WHO BMI chart for age (21–23). Each participant was put into the respective group based on his or her age and gender compared to the chart given.

### Sweet perception test

The method for recording the sweet taste threshold (the level at which a subject is able to detect the presence of sucrose in the solution) and preference (the preferred sucrose level) was adapted from Furquim et al. (24), according to the method originally used by Nilsson and Holm (25) and Zengo and Mandel (26). Ten sucrose solutions, ranging from 1.63 g/L (0.0047 M/L) to 821.52 g/L (2.40 M/L), were offered to the participants individually in order of increasing concentration and served in 10 mL disposable plastic medicine cups. The author and co-author of the study were present to assist with the tasting assessment. The participants were asked to circulate each of the tested solutions throughout their mouths for 5–10 sec and then spit, after which they were asked to identify their sweet taste threshold level and indicate the preferred solution. The children were allowed to make repeated tests of a solution if necessary. Two minutes were allowed between tasting the different test solutions. The solutions were prepared by the examiner the same day and served at room temperature.

### Caries registration

For caries assessment, ICDAS (International Caries Detection and Assessment System) and DMFS (Decayed Missing Filled Surfaces) indices were used. The ICDAS is a clinical scoring index used for diagnosing caries. The second digit number in ICDAS represents the caries component of the tooth, and depending on the tooth caries status it is scored from 1 to 6. The ICDAS-registered carious lesions 1, 2, and 3 affecting the enamel were considered as caries involving the enamel, while the lesions scored 4, 5, and 6 involved the dentine. Caries registration using the DMFS index followed the WHO criteria. In addition, DMFS is an index used for assessing dental caries prevalence, recording at dentinal level as number of decayed, missing, and filled teeth. Following the infection control guidelines, masks and gloves were used. Children were examined in their school setting under natural light using a dental probe and mirror from disposable dental examination kits (19).

### Statistical analysis

The mean, standard deviation, and range for the tested variables (taste threshold, taste perception, BMI, DMFS) were analyzed using IMB^®^ SPSS^®^ (PASW version 23.0 IBM^®^, Chicago, IL, USA). The difference between countries in terms of the studied variables was tested using ANOVA. A *p*-value of <0.05 was recorded as statistically significant. Spearman’s rank correlation was used for correlation analysis between variables. Differences between BMI groups (underweight, normal, overweight, and obese) were tested using ANOVA.

## Results

A statistically significant difference was found for BMI between children from Italy, Mexico, and Saudi Arabia (*p* < 0.001), with the highest values found for Saudi children (23.9 ± 6.1), followed by Mexican then Italian children (22.0 ± 3.4 and 20.6 ± 2.2, respectively). The range for BMI was also larger for children in Saudi Arabia (12.6–44.5), compared with the other two countries (14.9 ± 27.1 for Italy and 17.1 ± 35.2 for Mexico).

**Fig. 1:**
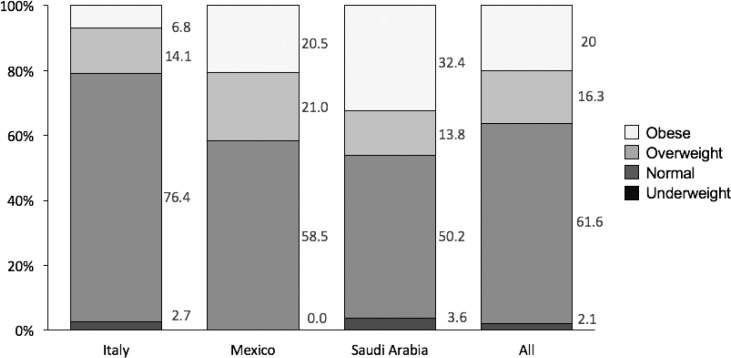
Frequency as a percentage for obese, overweight, normal weight, and underweight children in Italy, Mexico, and Saudi Arabia following the WHO recommendations for BMI for children (22) (*n* = 220, 224, and 225, respectively).

No statistical differences between countries were found for age or gender distribution (data not in table). A statistically significant difference between boys’ and girls’ mean BMI was found in Saudi Arabia (*p* < 0.01). However, in Italy and Mexico the BMI differences were numerical between boys and girls, with the higher mean BMI value among boys (ns). When participants were divided into BMI quartile groups, the prevalence of overweight and obesity was found to be higher among boys (Table 1). The mean BMI, all participants taken together, was 22.1. A total of 76.4% of the Italian children and 58.5% of the Mexican children were within the normal weight range, while the lowest figure was found for Saudi Arabian children (50.2%) (Fig. 1, Table 1). Of the Saudi children, 32.4% were found to be obese, compared with 20.5% in Mexico and 6.8% in Italy (Fig. 1, Table 1). The number of subjects (boys and girls) and percentage of BMI groups (underweight, normal, overweight, and obese) are shown in Table 1.

**Table 1:** Number and percentage of children divided into body mass index quartile groups in Italy, Mexico, and Saudi Arabia

BMI groups	Italy —————————			Mexico —————————			Saudi Arabia —————————		
	Boys (*n* = 110)	Girls (*n* = 110)	Total (*n* = 220)	Boys (*n* = 119)	Girls (*n* = 105)	Total (*n* = 224)	Boys (*n* = 114)	Girls (*n* = 111)	Total (*n* = 225)
Underweight count %	0 0	6 5.5	6 2.7	0 0	0 0	0 0	4 3.5	4 3.6	8 3.6
Normal count %	73 66.4	95 86.4	168 76.4	58 48.7	73 69.5	131 58.5	49 43.0	64 57.7	113 50.2
Overweight count %	25 22.7	6 5.5	31 14.1	33 27.7	14 13.3	47 21.0	11 9.6	20 18.0	31 13.8
Obese count %	12 10.9	3 2.7	15 6.8	28 23.5	18 17.1	46 20.5	50 43.9	23 20.7	73 32.4
Total count %	110 100	110 100	220 100	119 100	105 100	224 100	114 100	111 100	225 100

Italian children had the highest sweet taste threshold (65.0 ± 82.6), followed by Saudi (37.7 ± 47.5) and Mexican children (7.5 ± 4.4). Meanwhile, the highest values for sweet taste preference were seen for children from Saudi Arabia (319.69 ± 302.13), followed by Italian (231.88 ± 315.06) and Mexican children (25.4 ± 16.4). Children from Italy and Saudi Arabia had the largest ranges for both sweet taste preference and sweet taste threshold. However, for Mexican children, the range was small and none of the participants chose a solution of ≥51.35 gm/L.

The mean caries experience for Italian children was 1.7, 0.2, and 1.3 for DMFS, initial caries, and manifest caries, respectively. The corresponding figures for Mexico were 1.2, 0.8, and 1.2, respectively, and for Saudi Arabia 3.0, 6.7, and 1.3, respectively. The mean caries experience for all the subjects taken together was 2.0, 2.6, and 1.3 for DMFS, initial caries, and manifest caries, respectively.

No significant correlation was found between BMI and the sweet taste threshold or preference and dental caries variables, when the data for each country were tested separately. The strongest correlation was found for taste preference for the Italian children.

Taste perception and dental caries in relation to the different BMI groups (underweight, normal, overweight, and obese) are shown in Tables 2 and 3. No statistically significant difference was found for the tested variables between the BMI groups for the individual countries, with the exception of taste threshold in Saudi Arabia (*p* < 0.01).

**Table 2:** Mean and standard deviation for sweet taste threshold and sweet taste preference in children from Italy, Mexico, and Saudi Arabia, according to BMI groups (underweight, normal, overweight, and obese)

Variable	Underweight			Normal			Overweight			Obese				*p*-value^1^
	Mean ± SD	*n*	Range	Mean ± SD	*n*	Range	Mean ± SD	*n*	Range	Mean ± SD	*n*	Range		
**Italy**													
TT	55.6 ± 25.2	6	25.7–102.7	64.8 ± 69.3	168	3.3–410.8	88.1 ± 146.2	31	3.3–821.5	23.8 ± 18.6	15	3.3–51.3	0.100
TP	172.0 ± 322.0	6	1.6–821.5	213.5 ± 304.2	168	1.6–821.5	313.8 ± 353.2	31	1.6–821.5	291.9 ± 347.1	15	1.6–821.5	0.332
**Mexico**													
TT	–		–	7.6 ± 4.5	131	1.6–25.7	7.2 ± 4.8	47	1.6–25.7	7.4 ± 3.7	46	1.6–12.8	0.837
TP	–		–	26.6 ± 16.6	131	3.3–51.3	23.5 ± 17.1	47	3.3–51.3	24.2 ± 15.3	46	3.3–51.3	0.436
**Saudi Arabia**													
TT	116.3 ± 145.2	8	6.5–410.8	33.5 ± 40.5	113	1.6–205.4	27.3 ± 23.4	31	1.6–102.7	40.1 ± 37.9	73	1.6–205.4	<0.01
TP	388.3 ± 378.7	8	25.7–821.5	308.8 ± 297.1	113	1.6–821.5	262.5 ± 281.0	31	12.8–821.5	353.3 ± 310.7	73	12.8–821.5	0.465

1 *p*-value: Tests significance between BMI groups.

SD, standard deviation; TT, sweet taste threshold; TP, sweet taste preference.

**Table 3:** Mean and standard deviation for DMFS, initial, and manifest caries in Italy, Mexico, and Saudi Arabia according to BMI groups (underweight, normal, overweight, and obese)

Variable	Underweight			Normal			Overweight			Obese			*p*-value^1^
	Mean ± SD	*n*	Range	Mean ± SD	*n*	Range	Mean ± SD	*n*	Range	Mean ± SD	*n*	Range	
**Italy**													
DMFS	2.3 ± 2.3	6	0–6	1.6 ± 2.2	168	0–10	2.4 ± 3.5	31	0–13	0.7 ± 0.9	15	0–2	0.124
Initial	0.3 ± 0.08	6	0–2	0.2 ± 0.6	168	0–4	0.2 ± 0.6	31	0–3	0.2 ± 0.6	15	0–2	0.935
Manifest	2.0 ± 2.4	6	0–6	1.3 ± 1.9	167	0–8	1.9 ± 3.0	30	0–11	0.5 ± 0.8	15	0–2	0.141
**Mexico**													
DMFS	–		–	1.3 ± 1.6	131	0–6	1.2 ± 1.7	47	0–7	1.0 ± 1.3	46	0–4	0.676
Initial				0.9 ± 1.4	131	0–6	0.8 ± 1.4	47	0–7	0.8 ± 1.2	46	0–4	0.905
Manifest	–		–	1.2 ± 1.5	131	0–6	1.1 ± 1.6	47	0–7	1.0 ± 1.3	46	0–4	0.807
**Saudi Arabia**													
DMFS	3.3 ± 4.7	8	0–14	2.8 ± 3.7	113	0–22	3.5 ± 4.7	31	0–23	2.9 ± 1.4	73	0–25	0.873
Initial	6.0 ± 5.3	8	0–12	7.7 ± 9.4	113	0–59	5.6 ± 6.9	31	0–34	5.9 ± 5.4	73	0–21	0.368
Manifest	0.5 ± 1.1	8	0–3	1.1 ± 2.1	113	0–14	1.8 ± 3.6	31	0–17	1.6 ± 3.5	73	0–24	0.448

1 *p*-value: Tests significance between BMI groups.

SD, standard deviation; DMFS, Decayed Missing Filled Surfaces.

## Discussion

The main finding from this study is the statistically significant difference for BMI in participating 13–15-year-old children from Italy, Mexico, and Saudi Arabia. The differences may be a result of variations in dietary habits, lifestyle including physical activity, environmental influence, and genetic factors.

It is believed that the higher proportion of obese children in Saudi Arabia in comparison to Mexico and Italy can be primarily attributed to dietary factors. In Saudi, more than 90% of schoolchildren are known to consume sweets regularly (27). Furthermore, both high sweet consumption and unhealthy dietary habits have previously been reported among Saudi children and adolescents (28, 29). There is accumulating evidence suggesting that Mediterranean lifestyles, including nutrition and sleeping patterns, as well as social integration, may play a role in reducing age-related diseases. In Italy, a recent study described how young Italian students encountered considerable difficulties in conducting a healthy lifestyle, doing little physical activity and acquiring unfavorable dietary habits (30).

Despite the unanticipated low percentage of obese Italian children in our findings, a study by Saulle, Del Prete (31) stated that more than 63% of the adolescent group in Italy do not follow the healthy dietary recommendations encompassed by the Mediterranean diet. Two important characteristics of the Mediterranean diet, low consumption of saturated fatty acids and high intake of carbohydrates, are missing. In addition, Wijnhoven, van Raaij (32) reported that Italian children have a higher rate of obesity than stated in our findings. However, a study by Mistura, D’Addezio (33) in Italy stated that the consumption of unhealthy, high caloric intake soft drinks was only 0.4% of the total daily intake. In Mexico, a finding similar to that in our study was reported by Gutierrez-Pliego, Camarillo-Romero Edel (34), where it was found that 67% of the adolescents were within the normal range of BMI, 24.3% overweight, and 8.5% obese. Children in Mexico are known to usually skip breakfast, which is a known indicator of more unhealthy dietary habits (34). However, more than 50% of Mexican children have been reported to participate in some sort of physical activity at least twice a week (35). Although not evaluated in this study, a high energy intake and less physical activity, aggravated by modern technologies, may have an effect on the final energy balance (4).

The use of BMI for anthropometrical measurements has been discussed (21, 22, 36), but it has to be regarded as a system that is easy to perform as a chairside method. Furthermore, the actual cutoff values to be used to categorize the individuals can also be questioned, particularly considering the age of the participants (36). The handling of the data followed the WHO recommendations for BMI for age and gender in children (23). The age group included was selected to assure that the children were old enough to be in the permanent dentition phase and could give an accurate description of their sweet taste perception.

Only a few studies have so far been published regarding the relationship between sweet taste and BMI or obesity (14, 37). No association between sweet food and overweight has previously been found (37), in accordance with our findings. In contrast, the study by Lanfer, Knof (14) reported a relationship between obesity and sweet preference.

In the present study, no correlation was found between BMI and caries. However, a study by Quadri, Hendriyani (27) reported a correlation between BMI and caries, especially in German children from high socioeconomic groups, which may be a result of the high sugar intake in this group. Although not examined in the present study, a higher risk of dental erosion compared with dental caries among obese children has also recently been reported (38). From what can be concluded, a higher sugar intake may contribute to caries and obesity (39), and special consideration is called for to lead the children’s sense of taste to healthier habits for eating.

Taste has been found to be more pronounced in normal-weight than obese children and a higher threshold for sweet taste among obese children was therefore reported (10, 40). In the present study, differences of sweet taste threshold were found between BMI groups in Saudi children. These differences were not found when analyzing the data in Mexico and Italy. This may be explained by the overall limited number of participants, which resulted in a small number of subjects in some categories. For example, in Italy, there were only six subjects in the underweight group, while there were none in Mexico and only eight in Saudi Arabia. This also applies to the obese group in Italy, with only 15 subjects, where most of the study subjects were found within the normal-weight group.

A higher DMFS value was found among the underweight group of children for all subjects. This finding may be interpreted as the caries status affecting the BMI, where children can experience difficulty in eating because of the presence of caries (41). In addition, Norberg, Hallström Stalin (41) suggested that there may be concerns regarding parental influence on diet for underweight children, and thus they may be exposed to foods that have a negative effect on their teeth.

In conclusion, the BMI in total was found to differ between countries; the exact reason for this has not been addressed in this study and thus remains unresolved. The present findings should therefore be interpreted with care, because of the difference in cultural and environmental background in the three countries. In addition, the socioeconomic group recruited for this study was from the middle-income level. The possibility of obtaining different results from other socioeconomic levels cannot be excluded.

It is well known that setting up a multicenter study poses a number of challenges, including the identification of comparable test subjects. However, although this approach does present difficulties, it offers the potential to build a stronger scientific basis for the conclusions. It is important that multisite clinical trials be preceded by comprehensive planning, including standardization, training, and calibration.

It is necessary to stress that, even if the same BMI picture is presented, the exact causal factors may differ. There is still a need for studies focusing on the effect of sweets consumption on BMI, as well as dental caries, with special emphasis on the factors determining taste preference. As mentioned by Drewnowski (13), studies are needed to focus on the sensory factors affecting food preference and energy intake in diet to help design enhanced dietary plans and help control obesity. It has been suggested that it is important for future studies of obesity to include the ethnic background and socioeconomic status of participants (42). In addition, public health recommendations for appropriate dietary habits for both children and adults may have an impact on both general and oral health, and interest needs to focus on both areas.
